# Clinical presentation and hospitalisation duration of 201 coronavirus disease 2019 patients in Abuja, Nigeria

**DOI:** 10.4102/phcfm.v13i1.2940

**Published:** 2021-10-27

**Authors:** Isaac O. Akerele, Adaeze C. Oreh, Mohammed B. Kawu, Abubakar Ahmadu, Josephine N. Okechukwu, Danjuma N. Mbo, Doris J. John, Faridah Habib, Matthew A. Ashikeni

**Affiliations:** 1 Department of Family Medicine, Asokoro District Hospital COVID-19 Isolation and Treatment Centre, Federal Capital Territory Administration, Abuja, Nigeria; 2 Department of Planning, Research and Statistics, National Blood Transfusion Service, Federal Ministry of Health, Abuja, Nigeria; 3 Faculty of Health and Human Services Secretariat, Federal Capital Territory Administration, Abuja, Nigeria; 4 Department of Anaesthesia, Faculty of Health and Human Services Secretariat, Federal Capital Territory Administration, Abuja, Nigeria; 5 Department of Public Health, Faculty of Health and Human Services Secretariat, Federal Capital Territory Administration, Abuja, Nigeria; 6 Department of Infectious Diseases and Immunology, Maitama District Hospital, Federal Capital Territory Administration, Abuja, Nigeria; 7 Department of Family Medicine, Nisa Premier Hospital, Abuja, Nigeria

**Keywords:** coronavirus disease 2019, COVID-19, SARS-CoV-2, presentation, duration of hospitalisation, hospital stay, patient education, Abuja, Nigeria

## Abstract

**Background:**

Knowledge of severe acute respiratory syndrome coronavirus 2 (SARS-CoV-2) infection is unfolding. Insights from patient features in different environments are therefore vital to understanding the disease and improving outcomes.

**Aim:**

This study aimed to describe patient characteristics associated with symptomatic presentation and duration of hospitalisation in coronavirus disease 2019 (COVID-19) patients managed in Abuja.

**Setting:**

The study was conducted in Abuja, the Federal Capital Territory, Nigeria.

**Methods:**

This was a retrospective study of 201 COVID-19 patients hospitalised in the Asokoro District Hospital COVID-19 Isolation and Treatment Centre between April 2020 and July 2020. Demographic and clinical data were obtained and outcomes assessed were symptom presentation and duration of hospitalisation.

**Results:**

Patients’ median age was 39.3 years (interquartile range [IQR]: 26–52); 65.7% were male and 33.8% were health workers. Up to 49.2% of the patients were overweight or obese, 68.2% had mild COVID-19 at presentation and the most common symptoms were cough (38.3%) and fever (33.8%). Hypertension (22.9%) and diabetes mellitus (7.5%) were the most common comorbidities. The median duration of hospitalisation was 14.4 days (IQR: 9.5–19). Individuals with secondary and tertiary education had higher percentage symptoms presentation (8.5% and 34%, respectively), whilst a history of daily alcohol intake increased the length of hospital stay by 129.0%.

**Conclusion:**

Higher educational levels were linked with symptom presentation in COVID-19 patients and that daily alcohol intake was significantly associated with longer hospital stay. These findings highlight the importance of public education on COVID-19 for symptom recognition, early presentation and improved outcomes.

## Introduction

Since December 2019 when the coronavirus disease 2019 (COVID-19) was first reported from Wuhan, China, by the World Health Organization (WHO), numbers of infected cases have steadily continued to rise globally. As at the time of writing, over 107 million cases have been reported across the world, with more than 2 million deaths.^1^ In Nigeria, the first case was identified on 27 February 2020 and since then, there have been over 140 000 confirmed cases, 114 635 patients have been discharged and 1673 deaths have been recorded from the virus.^2^

Whilst Lagos State has been the epicentre of the outbreak in Nigeria (36.9% of the confirmed cases), Abuja the Federal Capital Territory (FCT) is the state with the second highest number of infections, accounting for 12.7% of the confirmed cases in the country.^2^

The severe acute respiratory syndrome coronavirus 2 (SARS-CoV-2) is transmitted via respiratory droplets, with current epidemiological evidence suggesting an incubation period of 5–14 days.^3,4^ Clinical presentation is said to be more predominant in males, with more common symptoms such as fever, dry cough, shortness of breath, headache, fatigue, sore throat, loss of smell, loss of taste and myalgia; in addition to less common symptoms such as nausea, vomiting and diarrhoea.^3^ Additional laboratory and radiographic findings include prolonged prothrombin time, lymphopenia and pulmonary infiltrates on chest radiography.^5^ The absence of symptoms as outlined here, however does not preclude viral transmission in asymptomatic and pre-symptomatic carriers.

The national strategy to fight COVID-19 which was adopted by Abuja, the FCT was the ‘Test, Trace, Isolate and Treat’. Asokoro District Hospital was the second hospital after the University of Abuja Teaching Hospital, Gwagwalada, designated for the management of COVID-19 cases in Abuja that received a significant number of confirmed cases of COVID-19. To adequately implement a successful response to COVID-19 in Nigeria, a clear understanding of the demographic and clinical profiles of COVID-19 in the local environment is needed.^6^

Several studies documenting the spectrum of clinical characteristics of COVID-19 patients in South-West Nigeria are available.^6,7,8^ However, evidence from Northern Nigeria is not readily available. As the COVID-19 pandemic continues to spread rapidly, with countries recording new waves of heightened infections, it is pertinent that locally derived evidence adds to the body of knowledge and is used to strengthen national and global public health responses to the pandemic.

This study examines the sociodemographic and clinical characteristics of the first 201 laboratory-confirmed polymerase chain reaction (PCR) positive COVID-19 patients treated at Asokoro District Hospital in Abuja Nigeria. Furthermore, it relates these features to outcomes such as symptom presentation and the duration of hospitalisation for COVID-19 and identifies lessons to reduce transmission of the virus and improve patient outcomes.

## Methodology

### Study design

This was a retrospective study of 201 cases managed at the Asokoro COVID-19 Isolation and Treatment Centre in Asokoro District Hospital, Abuja, between 10 April 2020 and 31 July 2020. This study forms part of the Asokoro District Hospital COVID-19 Clinicopathological Profile Project, which investigates the clinical, laboratory, radiologic and pathologic presentation of COVID-19 patients managed at the hospital.

### Study settings

The study was conducted on COVID-19 patients treated at the COVID-19 Isolation and Treatment centre in Asokoro District Hospital, Abuja, the FCT, North-Central Nigeria. According to the 2018 National Demographic and Health Survey, Abuja has a population of over 3.5 million.^9,10^ The Asokoro District Hospital, being one of the foremost public infectious disease hospitals in Nigeria FCT, was the second facility in Abuja designated to isolate and treat COVID-19 patients with funding support from the WHO, the United States Centers for Disease Control and Prevention (US CDC), and African Field Epidemiology Network (AFENET). It has a 60-bed admission treatment facility with a female to male bed space ratio of 20:40, a dedicated biosafety laboratory, emergency operations centre, and a biosecurity unit.

The process for admission entailed identification of laboratory-confirmed SARS-CoV-2 individuals by a remote triage team, coordinated by the State Epidemiologist and Department of Public Health followed by transportation of the confirmed individuals to the facility in a COVID-19 evacuation ambulance. On arrival at the isolation facility, consent was obtained and baseline clinical assessments were conducted after which a bedspace was allocated to individuals who accepted inpatient care. Discharge from the isolation facilities during the period of the study was based on the WHO criteria: (1) three days after resolution of symptoms and (2) two negative Reverse transcription polymerase chain (RT-PCR) SARS-CoV-2 results, at least 24 h apart.^11,12^

### Study data

Case records of hospitalised and outpatient laboratory-confirmed COVID-19 cases seen between 10 April 2020 and 31 July 2020 were obtained. Inclusion criterion for the selected records was a laboratory-confirmed SARS-CoV-2 infection. A laboratory-confirmed case of the SARS-CoV-2 virus was defined as a positive result on high–throughput sequencing or real-time RT-PCR assay of a nasopharyngeal swab and oropharyngeal specimens based on the WHO guidelines.^13^

### Data collection tool

Patients’ demographic information, clinical symptoms and signs, comorbidities, laboratory and radiographic findings, in addition to information on the duration of hospitalisation till a negative COVID-19 test result were recorded. These medical records were then independently reviewed by a team of experienced clinicians who in addition to having 15 years’ of experience had also received extensive training in quality data management and abstracted data of cases seen during the study period. The abstracted data were collected from electronic medical records and entered in a Microsoft Excel spreadsheet (2019) under the coordination of the Research Unit of the FCT Health and Human Services Secretariat. Double entry was required for all variables and the data were reconciled by a third party. When missing data were encountered, requests for clarification were sent to the Case Manager at the COVID-19 treatment centre who subsequently contacted the attending clinicians or verified from the source documents. Of a total of 206 discharged cases as at 31 July 2020 (Nigeria Centre for Disease Control [NCDC] update week 31),^14^ 5 cases with missing or incomplete records on demographic and/or clinical information were excluded. There were no patient mortalities recorded.

### Study variables

The study outcomes of interest were the duration of hospitalisation (measured in days) and self-reported presence of symptoms from the time of laboratory diagnosis of COVID-19 and subsequent isolation at a health facility. Furthermore, the self-reported presence of comorbidities was assessed categorically: no comorbidity, one comorbidity and two or more comorbidities. The COVID-2019 severity was defined as mild, moderate or severe according to the US CDC definitions.^15^ Mild illness was defined as individuals who had any of the various signs and symptoms of COVID-19 (such as fever, cough, sore throat, malaise, headache, muscle pain, nausea, vomiting, diarrhoea, loss of taste and smell), but who did not have shortness of breath, dyspnoea or abnormal chest imaging. Moderate COVID-19 described individuals who showed evidence of lower respiratory disease during clinical assessment or imaging and who had an oxygen saturation (SpO_2_) ≥ 94% on room air at sea level. Severe illness was defined as individuals who had SpO_2_ < 94% on room air at sea level, a ratio of arterial partial pressure of oxygen to fraction of inspired oxygen (PaO2/FiO2) < 300 millimetre of mercury (mmHg), respiratory frequency > 30 breaths/min or lung infiltrates > 50%.^15^

### Statistical analysis

Statistical analysis was performed using International Business Machine Corporation (IBM) Statistical Package for the Social Sciences (SPSS) Statistics for Windows, version 24.0. (Armonk, NY: IBM Corp). Demographic information, medical history and COVID-19 symptoms were analysed using descriptive statistics. Pearson’s Chi-square test was used to determine an association between the categorical data. Bivariate and multivariate logistic regression models were used to investigate the association between demographic information, comorbidities (self-reported and determined following clinical assessment), the presence or absence of COVID-19 symptoms , and duration of hospitalisation. A logistic regression model was used to investigate the association between demographic information, number of comorbidities (none, 1 comorbidity and ≥ 2 comorbidities), symptomatic presentation and duration of hospitalisation (≤ 14 days and > 14 days). Tests of normality were used to assess continuous variables. A *p-*value < 0.05 was considered statistically significant and tests were 2–tailed with confidence levels of 95%.

## Results

### Demographic and clinical characteristics of patients

A total of 201 cases were analysed for this study, with a mean age of 39.3 years (standard deviation [s.d.] ± 13.1) and 65.7% (*n* = 132) of the patients male.

**FIGURE 1:**
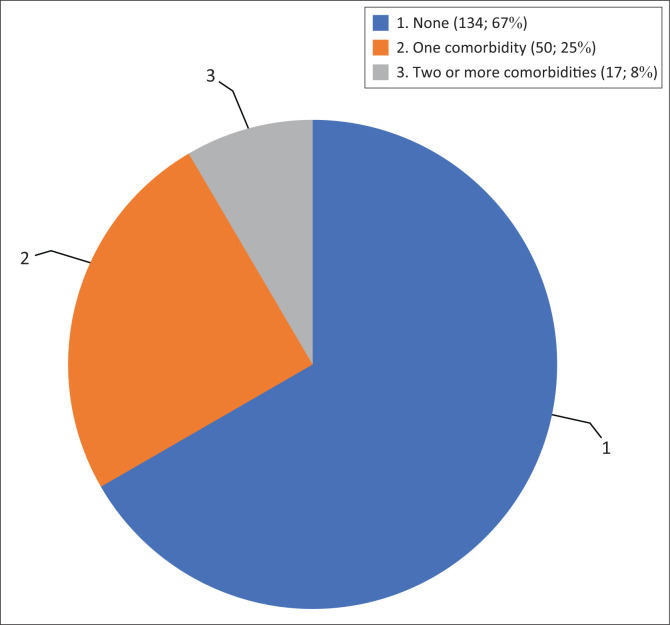
Number of comorbidities in patients admitted at Asokoro District Hospital Coronavirus Disease 2019 Isolation and Treatment Centre.

Approximately one-third of the patients (33.3%) presented with one or more comorbidities (Figure 1). Hypertension (22.9%), diabetes mellitus (7.5%), peptic ulcer disease (5.5%) and asthma (4.0%) were the most common comorbidities, whereas comorbidities such as liver disease, stroke and mental health disorders were less common. About 68.2% of the patients presented with a mild form of the disease and only 26.8% were asymptomatic at presentation. Out of all the patients studied, only 3.0% had severe disease and were in respiratory distress. The most common symptoms at presentation were: cough (38.3%), fever (33.8%), fatigue (26.9%), poor appetite (26.9%) and headache (25.4%). Insomnia (3.1%) and nausea (0.5%) were the least common symptoms. The mean duration of hospitalisation was 14.4 days (s.d. ± 4.9), with median duration of 13 days and 99% of the patients were discharged following management with 1% (*n* = 2) referral following diabetic complication of grade 4 diabetic foot ulcer requiring further specialist care than the facility could provide at that time and personal preference for another facility, respectively.

**FIGURE 2:**
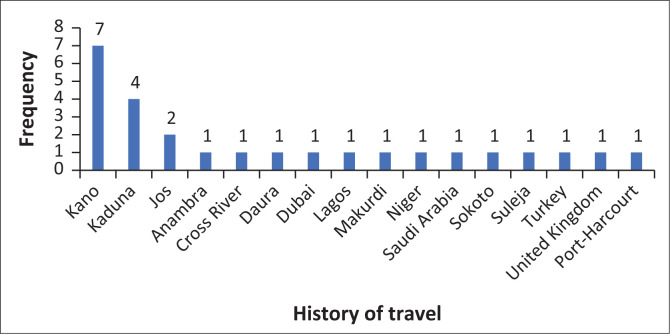
Chart depicting domestic and international travel history of COVID-19 patients managed in Asokoro, Abuja.

Overweight and obesity were observed in 49.2% (*n* = 99) of the patients, 2.0% had a known history of tuberculosis, 86.5% had a history of Bacillus Calmette-Guérin (BCG) vaccination, 12.9% had a recent travel history and the location of exposure was unknown in 69.7% of the patients. However, for those patients who knew their location of exposure (*n* = 61), the non-hospital workplace (34.4%) was the commonest location of exposure, closely followed by home (32.8%) and hospital (24.6%). International travel was observed in only 15.4% of the patients who gave a history of recent travel, as domestic travel accounted for 84.6% of the patients’ travels (Figure 2).

### Association between the COVID-19 patient demographic characteristics, comorbidity and symptomatic presentation

The association between patient demographic characteristics, comorbidity and symptomatic presentation is illustrated in Table 1. Age (*p* = 0.046), occupation (*p* = 0.015), employment status (*p* = 0.004) and education (*p* = 0.001) were significantly associated with the presentation of symptoms in patients. The relationship between possible risk factors such as body mass index (BMI), history of travel, history of tuberculosis and BCG vaccination, contact history, daily alcohol intake, smoking and patient symptoms were assessed. Only daily alcohol intake showed any association with symptomatic patient presentation (*p* = 0.024).

**TABLE 1:** Chi-square analysis to demonstrate the association between patient demographic factors and symptomatic COVID-19.

Variables	Symptomatic presentation				*χ* ^2^	*p*
	Asymptomatic (*n* = 54)		Symptomatic (*n* = 147)			
	*n*	%	*n*	%
**Age group (years)**					11.312	0.046*
< 20	7	13.0	4	2.7	-	-
20–29	11	20.4	24	16.3	-	-
30–39	16	29.5	38	25.9	-	-
40–49	11	20.4	36	24.5	-	-
50–59	7	13.0	36	24.5	-	-
≥ 60	2	3.7	9	6.1	-	-
**Gender**					0.681	0.409
Female	33	61.1	99	67.3	-	-
Male	21	38.9	48	32.7	-	-
**Occupation**					5.976	0.015*
Healthcare workers	11	20.4	57	38.8	-	-
Non-healthcare workers	43	79.6	90	61.2	-	-
**Employment status**					8.504	0.004*
Employed	46	85.2	142	96.6	-	-
Unemployed	8	14.8	5	3.4	-	-
**Education**					29.012	< 0.001*
No formal education	8	14.8	7	4.8		-
Primary	8	14.8	3	2.0	-	-
Secondary	16	29.7	23	15.6	-	-
Tertiary	22	40.7	114	77.6	-	-
**Comorbidity**					4.103	0.129
None	42	77.7	92	62.6	-	-
One	9	16.7	41	27.9	-	-
Two or more	3	5.6	14	9.5	-	-

* , Significant at *p* < 0.05.

Logistic regression was subsequently carried out to identify factors predictive of symptomatic COVID-19 presentation (Table 2). Patients with secondary and tertiary education showed significantly higher odds of presenting with symptoms of COVID-19 (odds ratio [OR]:1.085; with 95% confidence interval [CI]: 1.020–1.374; *p* = 0.001) and (OR: 1.340; with 95% CI: 1.137–1.847; *p* = 0.021), respectively, which translated to 8.5% and 34% increments, respectively, compared with patients with no formal education.

**TABLE 2:** Logistic regression model demonstrating the association between patient characteristics and symptomatic presentation.

Variables	*p*	Odds ratio	95% CI for odd ratio	
			Lower	Upper
**Age**				
< 20	1.000	(Reference)	-	-
20–29	0.813	0.732	0.056	9.652
30–39	0.828	0.821	0.138	4.880
40–49	0.775	0.780	0.141	4.316
50–59	0.791	1.271	0.217	7.452
≥ 60	0.749	1.348	0.216	8.407
**Occupation**				
Healthcare worker	1.000	(Reference)	-	-
Non-healthcare worker	0.733	1.169	0.477	2.867
**Employment status**				
Employed	1.000	(Reference)	-	-
Unemployed	0.549	1.778	0.271	11.671
**Education**				
No formal education	1.000	(Reference)	-	-
Primary	0.088	0.299	0.074	1.198
Secondary	0.001*	1.085	1.020	1.374
Tertiary	0.021*	1.340	1.137	1.847
**Daily alcohol intake** †				
No	1	(Reference)	-	-
Yes	0.112	2.053	0.846	4.985

CI, confidence interval.

* , Significant at *p* < 0.05.

† , Daily alcohol intake: Anyone who consumes one or more alcoholic drinks daily.^39^

### Association between the COVID-19 patient demographic characteristics and comorbidity and duration of hospitalisation

Table 3 illustrates the association between patient demographic characteristics and the duration of hospitalisation for COVID-19. Occupation (*p* < 0.001), education (*p* = 0.001) and comorbidity (*p* = 0.001) were significantly associated with the duration of hospitalisation. In Table 4, a significant association was demonstrated between BMI (*p* < 0.001), location of exposure (*p* = 0.020), daily alcohol intake (*p* = 0.001), contact history (*p* = 0.014), and the duration of hospitalisation for COVID-19. However, following logistic regression analysis (Table 5), only patients with a history of daily alcohol intake showed significantly higher odds of having a lengthened duration of hospitalisation (OR: 2.288; with 95% CI: 2.103– 2.804; *p* = 0.017), which translated to a 129% increment in length of hospital stay.

**TABLE 3:** Chi-square analysis demonstrating the association between patient demographic factors, comorbidity and duration of hospitalisation.

Variables	Duration of hospitalisation (days)				*χ* ^2^	*p*
	≤ 14 (*n* = 135)		> 14 (*n* = 66)			
	*n*	%	*n*	%
**Age group (years)**					9.521	0.090
< 20	6	4.4	5	7.5	-	-
20–29	17	12.6	18	27.3	-	-
30–39	36	26.7	18	27.3	-	-
40–49	35	25.9	12	18.2	-	-
50–59	32	23.7	11	16.7	-	-
≥ 60	9	6.7	2	3.0	-	-
**Gender**					3.202	0.074
Male	83	61.5	49	74.2	-	-
Female	52	38.5	17	25.8	-	-
**Occupation**					15.317	<0.001*
Healthcare workers	58	43.0	10	15.2	-	-
Non-healthcare workers	77	57.0	56	84.8	-	-
**Education**					17.391	0.001*
No formal education	7	5.2	8	12.1	-	-
Primary	4	3.0	7	10.6	-	-
Secondary	20	14.8	19	28.8	-	-
Tertiary	104	77.0	32	48.5	-	-
**Comorbidity**					13.430	0.001*
None	79	58.5	55	83.3	-	-
One	40	29.6	10	15.2	-	-
Two or more	16	11.9	1	1.5	-	-

* , Significant at *p* < 0.05.

**TABLE 4:** Chi-square analysis demonstrating the association between likely patient risk factors and duration of hospitalisation.

Variables	Duration of hospitalisation (days)				*χ* ^2^	*p*
	≤ 14 (*n* = 135)		> 14 (*n* = 66)			
	*n*	%	*n*	%
**Body mass index (BMI)**					33.696	< 0.001*
Normal	51	37.8	46	69.7	-	-
Obese	32	23.7	5	7.6	-	-
Overweight	52	38.5	10	15.2	-	-
Underweight	0	0.0	5	7.6	-	-
**History of tuberculosis**					3.290	0.105
No	134	99.3	63	95.5	-	-
Yes	1	0.7	3	4.5	-	-
**History of BCG vaccination**					3.268	0.659
No	7	5.2	6	9.1	-	-
Yes	127	94.8	60	90.9	-	-
**History of travel**					0.058	0.810
No	117	86.7	58	87.9	-	-
Yes	18	13.3	8	12.1	-	-
**Location of exposure**					11.680	0.020*
Not known	85	63.0	55	83.3	-	-
Workplace	16	11.8	5	7.6	-	-
Home	15	11.1	5	7.6	-	-
Hospital	15	11.1	0	0.0	-	-
Others†	4	3.0	1	1.5	-	-
**Smoking status** ‡					0.576	0.479
No	130	96.3	62	93.9	-	-
Yes	5	3.7	4	6.1	-	-
**Daily alcohol intake** §					10.273	0.001*
No	90	66.7	58	87.9	-	-
Yes	45	33.3	8	12.1	-	-
**Contact history**					10.665	0.014*
Not known	88	65.2	56	84.9	-	-
Confirmed patient	12	8.8	0	0.0	-	-
Colleague	9	6.7	2	3.0	-	-
Relative	26	19.3	8	12.1	-	-

BCG, Bacillus Calmette-Guérin.

Body mass index (BMI) values^36^: Underweight = < 1 8.5 kg/m^2^, normal weight = 18.5 kg/m^2^ – 24.9 kg/m^2^, overweight = 25 kg/m^2^ – 34.9 kg/m^2^, Obese = > 35 kg/m^2.^

* , Significant at *p* < 0.05.

† , Place of worship, police station and social gatherings;

‡ , anyone who has smoked at least 100 cigarettes in his or her lifetime and who now smokes every day OR anyone who has smoked at least 100 cigarettes in his or her lifetime but who had quit smoking at the time of interview (every-day smoker or former smoker)^39^;

§ , anyone who consumes one or more alcoholic drinks daily.^40^

**TABLE 5:** Logistic regression model demonstrating the association between patient characteristics and duration of hospitalisation.

Variables	*p*	Odds ratio	95% CI for odd ratio	
			Lower	Upper
**Occupation**				
Healthcare workers	1.000	(Reference)	-	-
Non-healthcare workers	0.081	0.420	0.159	1.112
**Education**				
No formal education	1.000	(Reference)	-	-
Primary	0.859	0.881	0.218	3.564
Secondary	0.584	1.512	0.345	6.624
Tertiary	0.749	1.178	0.431	3.221
**Comorbidity**				
None	1.000	(Reference)	-	-
One	0.083	6.698	0.777	57.712
Two or more	0.121	6.054	0.623	58.790
**Body mass index (BMI)**				
Normal	1.000	(Reference)	-	-
Obese	0.999	0.000	0.000	-
Overweight	0.999	0.000	0.000	-
Underweight	0.999	0.000	0.000	-
**Location of exposure**				
Not known	1.000	(Reference)	-	-
Workplace	0.942	1.126	0.046	27.611
Home	0.709	1.987	0.054	73.352
Hospital	0.892	1.293	0.032	51.729
Others†	0.998	0.000	0.000	-.
**Daily alcohol intake** ‡				
No	1.000	(Reference)	-	-
Yes	0.017*	2.288	2.103	2.804
**Contact history**				
Not known	1.000	(Reference)	-	-
Confirmed patient	0.747	1.829	0.047	71.517
Colleague	0.998	0.000	0.000	-
Relative	0.671	0.592	0.053	6.657

CI, confidence interval.

Body mass index values^36^: Underweight = < 18.5 kg/m^2^, normal weight = 18.5 kg/m^2^ – 24.9 kg/m^2^, overweight = 25.0 kg/m^2^ – 34.9 kg/m^2^, obese = > 35.0 kg/m^2^ – 34.9 kg/m^2^.

* , Significant at *p* < 0.05.

† , Place of worship, police station and social gatherings;

‡ , anyone who consumes one or more alcoholic drinks daily.^40.^

## Discussion

This study aimed at identifying the demographic characteristics and clinical profiles of a cohort of COVID-19 patients managed at the Asokoro COVID-19 Isolation and Treatment facility in Abuja, the FCT, Nigeria.

The study’s findings revealed a mean age of just below 40 years and that majority of patients were male. About 7 out of every 10 patients had symptoms present, approximately a third had one or more comorbidities and the mean duration of hospitalisation recorded was about 2 weeks. Higher levels of education were significantly associated with symptomatic presentation, whilst daily alcohol intake was significantly associated with the duration of hospitalisation.

The lower mean age recorded in this study is reflective of the younger population age in Nigeria compared with reports on the COVID-19 pandemic from the United Kingdom, Italy, United States and China.^9,10,16,17,18^ One major finding from our study is the preponderance of COVID-19 in economically active age groups, which points at the possible role of socio-economic or work-related activities in the transmission of the infection.

In this study, nearly 7 out every 10 patients had no idea where they may have been exposed to the SARS-CoV-2 virus. For those who knew their location of exposure, the workplace and the home accounted for about 67% and hospitals for healthcare workers accounted for a quarter of infections. This finding tallies with reports from research led by the Harvard T.H. Chan School of Public Health, which documented the substantial role of workplaces in facilitating spread of the disease during the early stages of the outbreak in six Asian countries.^19^ The Harvard study similarly demonstrated that non-hospital work-related transmissions were considerably more frequent. This emphasises the importance of ensuring strict adherence to COVID-19 preventive protocols such as wearing of face masks, physical distancing measures, and frequent hand washing and disinfection of surfaces in workplaces.^20^ The proportion of health workers infected (33.8%) in this study is substantially greater than earlier reports from Nigeria (9%)^21^ and has considerable implications for the already stretched healthcare workforce in fragile, under-resourced health systems. Further studies assessing infection rates with health facility preparedness in terms of availability of personal protective equipment (PPE), facility design, space management and cross-ventilation are therefore warranted. Space and ventilation issues are also relevant to COVID-19 transmission in non-hospital workplace environments.^22^

As this study was conducted during restrictions on places of worship and social gatherings, these accounted for less than 10% of patients. However, lapses in adherence to preventive measures always leave household members vulnerable, as nearly 33% of the patients were infected in their homes. Male predominance in presentation has similarly been reported in prior studies in Nigeria and globally.^20^ As women make up a substantial proportion of the workforce in Nigeria, it is worth mentioning the likely impact of the significant differences in membrane-bound angiotensin-converting enzyme 2 (mACE2) expression between males and females in the presentation of COVID-19.^23,24^

International travel restrictions were in place during the study but in the less than 15% of the patients who reported a history of travel, more than three-quarters of those trips were between states (Figure 2). It is therefore paramount that travellers continue to adhere to infection prevention measures and the public remains vigilant to the fact that a pandemic is ongoing, as anyone could be infected, especially given the fact that infections could be transmitted by asymptomatic or pre-symptomatic individuals.

Approximately 30% of the patients were asymptomatic at admission, which is almost double the number reported by other Nigerian researchers in the early phase of the pandemic.^6^ Nigeria’s Presidential Task Force on COVID-19 mandated isolation and treatment of positively tested cases in designated centres, which ensured that individuals who tested positive but may not have experienced any symptoms were thus admitted for treatment. Whilst earlier studies from Hong Kong and South Korea reported lower rates of asymptomatic cases in treatment centres, asymptomatic cases reported from South-West Nigeria were up to 60%.^7,25,26^ However, patients’ mean age and proportion of patients with comorbidities in our study were similar to those reported from Lagos State.^7^

Cough, fever and fatigue were the most predominant symptoms reported in this study, similar to findings from Zhejiang province in China.^27^ Our finding of significant association between higher levels of education and presentation of symptoms in patients may account for some differences in the reporting of symptoms. This highlights the importance of risk communication and education of the public via multiple channels on transmission and symptoms of COVID-19 to heighten public awareness. It is likely that higher levels of education expose individuals to more sources of information on COVID-19, thus enabling them to report symptoms more readily than those with lower levels of education. This is especially important, as any of the most common symptoms identified in our study could easily be passed off as symptoms of flu, malaria and even allergies. In this study, comorbidity was not significantly associated with symptomatic presentation following logistic regression, at variance with findings reported from Lagos, South-West Nigeria.^7^

The median duration of hospitalisation in this study was about 14 days, less than that reported by the NCDC, where the median length of stay of 111 patients was 19 days.^21^ However, both durations are within the range reported from studies outside of China (4–21 days), although lower in range compared with findings documented from China (4–53 days).^28^ These variations in duration of hospitalisation may be because of the differences between the Chinese National Health Commission management guidelines and the NCDC and WHO guidelines used in Nigeria, such as the use of traditional Chinese medicine for COVID-19, in addition to timing of the pandemic across countries.^11,12,21,29,30^

Another significant finding from this study was the association between self-reported daily alcohol intake and longer duration of hospitalisation. Several authors have documented the effect of alcohol on both increasing susceptibility to infection and its negative impact on nearly all lung cells and the integrity of the lung barrier function.^31,32^ In fact, heavy alcohol use was acknowledged as a risk factor for poor outcomes during the influenza pandemic of 1918.^31,33^ Despite this, the effect of alcohol on outcomes in the present COVID-19 pandemic has not been reported much by researchers and therefore given the increased propensity for alcohol use during periods of stress,^34,35^ this finding has important implications for the content of public health advisory as escalating COVID-19 infections are recorded in various locations.

In our study, nearly half of the patients studied were overweight or obese. However, this was not found to be significantly associated with symptom presentation or duration of hospitalisation. The US CDC and various research studies report that adults with excess weight are at a greater risk of illness severity, hospitalisation, impaired immune function, ventilation difficulties and death from COVID-19.^36,37^ This study’s failure to demonstrate significant associations between obesity, overweight and patient outcomes may require further investigation in larger cohorts of COVID-19 patients.

In addition, despite hypertension and diabetes mellitus being the most frequently observed comorbidities, similar to reports from Lagos State and China,^7,38^ comorbidity and age were not found to have significant association with duration of hospitalisation. This is contrary to findings reported from Lagos State and China, which cited older patients as having significantly higher odds of being hospitalised for longer durations.^7,38^ In this study, less than 6% of the patients were aged above 59 years, perhaps accounting for the lack of association observed between age, comorbidity and duration of hospitalisation. There are therefore implications of our findings for further studies exploring patient’s age, the duration of comorbidity, adherence to comorbidity treatment regimens and the extent of control and management in COVID-19 patients.

Almost all patients were discharged following management, contrary to many global reports that detail death rates between 15% and 37%.^16,17,18^ However, a report from the Zhejiang province of China just outside Wuhan, similarly reported discharge rates of 99%. In that study, the median age reported (41 years) was close to the median age of our study (40 years).^26^ Younger population ages with lower prevalence of comorbidities have been proffered as likely reasons for comparatively low death rates observed in Africa.

### Strengths and limitations

This study details information on a substantial number of COVID-19 cases managed in the early stage of the pandemic in Northern Nigeria and provides evidence that highlights the importance of COVID-19 preventive measures and policies. Furthermore, strengths of the study include a demonstration of factors that could be predictive of the presentation of COVID-19 symptoms in infected patients and the duration of hospitalisation of COVID-19 cases in Nigeria.

However, this study was subject to several limitations. Firstly, by studying only data from patients hospitalised and managed in the Asokoro Isolation and Treatment Centre in Abuja, the FCT, findings may not be generalisable to the entire country. Secondly, as a descriptive study, causal relationships cannot be clearly established. Also, there may have been some recall bias for symptoms and certain comorbidities in addition to social acceptability bias in patient reporting of certain lifestyle factors.

## Conclusion

This study examines the demographic and clinical profiles of a cohort of COVID-19 patients managed at the Asokoro COVID-19 Isolation and Treatment Facility in Abuja, the FCT, Nigeria. Less than 30% of the patients were asymptomatic and about 5% presented with a moderate to severe form of the disease. The commonest symptoms were cough, fever and fatigue and hypertension and diabetes mellitus were the most frequent comorbidities observed. Higher level of education was significantly associated with the presentation of symptoms in patients and daily alcohol intake was found to be significantly associated with the duration of hospitalisation.

These findings illuminate the importance of public education on risk factors and symptoms of the disease and lifestyle education to ensure early presentation, minimise illness and deaths from the infection, and optimise healthcare expenditure on the management of COVID-19.
